# Supplementary material for: Evaluation of Heterocyclic Carboxamides as Potential Efflux Pump Inhibitors in Pseudomonas aeruginosa
Source: Antibiotics (Basel). 2021 Dec 28;11(1):30. doi: 10.3390/antibiotics11010030 (PMC8772707; doi:10.3390/antibiotics11010030)
Supplement: Supplementary file 1 [file antibiotics-11-00030-s001.zip › antibiotics-1474102-supplementary.pdf]

<u>**Supplementary Materials**</u>

Evaluation of Heterocyclic Carboxamides as Potential Efflux Pump Inhibitors in *Pseudomonas aeruginosa*

Yi Yuan, Jesus D. Rosado-Lugo, Yongzheng Zhang, Pratik Datta, Yangsheng Sun, Yanlu Cao, Anamika Banerjee and Ajit K. Parhi *

TAXIS Pharmaceuticals, Inc., 9 Deer Park Drive, Suite J-15, Monmouth Junction, New Jersey 08852, USA
Correspondence: aparhi@taxispharma.com (A.K.P.); Tel.: +1 732-230-3074

Chemistry Supporting Information

All solvents and chemicals were used as purchased without further purification. Mass spectrometry (MS) was performed by electrospray (ESI) ionization using a Shimadzu 2020 LC-MS quadrupole mass spectrometer. ^{1}H and ^{13}C-NMR were recorded using a Varian Oxford 300, Chemical shifts are given in parts per million (ppm) (δ relative to residual solvent peak for ^{1}H and ^{13}C). The coupling constants (*J*) are reported in hertz (Hz).

All new compounds are prepared according to the general synthetic procedure and the spectral data is reported here:

(*S*)-*N*-(2,5-diaminopentyl)-5-(4-fluorophenyl)benzo[*d*]thiazole-2-carboxamide hydrogen chloride salt **6a**.

^{1}H NMR (300 MHz, DMSO-d$_6$) δ 9.32 (m, 1H), 8.30 (m, 1H), 8.29 (m, 1H), 8.22 (s br, 2H), 8.05 (s br, 2H), 7.88 (m, 1H), 7.83 (m, 2H), 7.35 (t, *J* = 9 Hz, 2H), 3.50-3.68 (m, 3H), 2.79 (m, 2H), 1.68 (m, 4H).

(*S*)-*N*-(2,5-diaminopentyl)-6-(4-fluorophenyl)benzo[*d*]thiazole-2-carboxamide hydrogen chloride salt **6b**.

^{1}H NMR (300 MHz, DMSO-d$_6$) δ 9.31 (m, 1H), 8.53 (s, 1H), 8.28 (s br, 2H), 8.16 (m, 1H), 8.13 (s br, 2H), 7.90 (m, 1H), 7.81 (m, 2H), 7.34 (t, *J* = 9 Hz, 2H), 3.46-3.66 (m, 3H), 2.79 (m, 2H), 1.69 (m, 4H). MS (ESI+) m/z: calcd for $C_{19}H_{22}FN_4OS$ [M+H]$^+$: 373.15; found 373.20.

(*S*)-*N*-(2,5-diaminopentyl)-6-(4-fluorophenyl)-1*H*-benzo[*d*]imidazole-2-carboxamide hydrogen chloride salt **6c** has been previously described by E. J. LaVoie et al. in patent application: WO2018,165,611.

(*R*)-*N*-(2,5-diaminopentyl)-6-(4-fluorophenyl)-1*H*-benzo[*d*]imidazole-2-carboxamide hydrogen chloride salt **6d** has been previously described by E. J. LaVoie et al. in patent application: WO2018,165,611.

(*S*)-*N*-(2,5-diaminopentyl)-5-(4-fluorophenyl)benzofuran-2-carboxamide hydrogen chloride salt **6e**

^{1}H NMR (300 MHz, D_2O) δ 8.23 (s, 1H), 7.71 (m, 3H), 7.66 (m, 2H), 7.20 (t, *J* = 9 Hz, 2H), 3.75 (m, 1H), 3.67 (m, 1H), 3.57 (m, 1H), 3.02 (m, 2H), 1.82 (m, 4H). MS (ESI+) m/z: calcd for $C_{20}H_{23}FN_3O_2$ [M+H]$^+$: 356.18; found 356.05.

(*S*)-*N*-(2,5-diaminopentyl)-6-(4-fluorophenyl)benzofuran-2-carboxamide hydrogen chloride salt **6f**

^{1}H NMR (300 MHz, D_2O) δ 7.81 (m, 2H), 7.72 (m, 2H), 7.61 (m, 1H), 7.55 (s, 1H), 7.24 (t, *J* = 9 Hz, 2H), 3.78 (m, 1H), 3.63 (m, 1H), 3.58 (m, 1H), 3.05 (m, 2H), 1.83 (m, 4H). MS (ESI+) m/z: calcd for $C_{20}H_{23}FN_3O_2$ [M+H]$^+$: 356.18; found 356.05.

(*S*)-*N*-(2,5-diaminopentyl)-5-(4-fluorophenyl)benzo[*b*]thiophene-2-carboxamide hydrogen chloride salt **6g**

^{1}H NMR (300 MHz, D_2O) δ 7.97 (s, 1H), 7.85 (m, 1H), 7.78 (s, 1H), 7.60 (m, 3H), 7.18 (m, 2H), 3.66 (m, 1H), 3.61 (m, 1H), 3.52 (m, 1H), 3.06 (m, 2H), 1.81 (m, 4H). MS (ESI+) m/z: calcd for $C_{20}H_{23}FN_3OS$ [M+H]$^+$: 372.15; found 372.00.

(*S*)-*N*-(2,5-diaminopentyl)-5-(4-fluorophenyl)benzo[*b*]thiophene-2-carboxamide hydrogen chloride salt **6h**

^{1}H NMR (300 MHz, D_2O) δ 7.96 (s, 1H), 7.84 (m, 1H), 7.82 (s, 1H), 7.60 (m, 3H), 7.18 (m, 2H), 3.64 (m, 1H), 3.60 (m, 1H), 3.51 (m, 1H), 3.04 (m, 2H), 1.81 (m, 4H). MS (ESI+) m/z: calcd for $C_{20}H_{23}FN_3OS$ [M+H]$^+$: 372.15; found 372.00.

(*S*)-*N*-(2,5-diaminopentyl)-5-(4-fluorophenyl)-1*H*-indole-2-carboxamide hydrogen chloride salt **6i** has been previously described by E. J. LaVoie et al. in patent application: WO2018,165,612.

(*R*)-*N*-(2,5-diaminopentyl)-5-(4-fluorophenyl)-1*H*-indole-2-carboxamide hydrogen chloride salt **6k** has been previously described by E. J. LaVoie et al. in patent application: WO2018,165,612.

(*R*)-*N*-(2,5-diaminopentyl)-6-(4-fluorophenyl)-1*H*-indole-2-carboxamide hydrogen chloride salt **6l** has been previously described by E. J. LaVoie et al. in patent application: WO2018,165,612

(*S*)-*N*-(2,5-diaminopentyl)-5-(4-fluorophenyl)-1*H*-pyrrolo[2,3-*c*]pyridine-2-carboxamide hydrogen chloride salt **6m**

^{1}H NMR (300 MHz, CD_3OD) δ 9.07 (s, 1H), 8.48 (s, 1H), 7.93 (m, 2H), 7.71 (s, 1H), 7.40 (t, *J* = 8.7 Hz, 2H), 3.83 (m, 1H), 3.68 (m, 1H), 3.52 (m, 1H), 3.04 (m, 2H), 1.89 (m, 4H). ^{13}C NMR (75 MHz, CD_3OD) δ 161.02, 143.04, 140.24, 138.53, 130.96, 129.94, 129.83, 129.74, 129.19, 128.47, 117.25, 116.40, 116.09, 104.17, 51.81, 40.63, 38.85, 27.14, 23.23. MS (ESI+) m/z: calcd for $C_{19}H_{23}FN_5O$ [M+H]$^+$: 356.19; found 356.20.

(*S*)-*N*-(2,5-diaminopentyl)-6-(4-fluorophenyl)-1*H*-pyrrolo[3,2-*c*]pyridine-2-carboxamide hydrogen chloride salt **6n**

^{1}H NMR (300 MHz, D_2O) δ 9.14 (s, 1H), 7.88 (s, 1H), 7.85 (m, 2H), 7.55 (s, 1H), 7.36 (t, *J* = 8.7 Hz, 2H), 3.82 (m, 1H), 3.69 (m, 1H), 3.59 (m, 1H), 3.04 (m, 2H), 1.82 (m, 4H). MS (ESI+) m/z: calcd for $C_{19}H_{23}FN_5O$ [M+H]$^+$: 356.19; found 356.25.

Table S1. Levofloxacin potentiation in the presence of EPI on *P. aeruginosa* strains overexpressing efflux pumps.

Compound	Strain				
	**K767 (WT)**	**K1455* (↑mexAB-oprM)**	**K2415* (↑mexXY-oprM)**	**K2951* (↑mexCD-oprJ)**	**K2376# (↑mexEF-oprN)**
	**Levofloxacin MIC (µg/mL), (fold difference)**				
**6f**	0.125, (4)	1, (4)	0.5, (4)	0.125, (4)	4, (2)
**6g**	0.063, (8)	0.25, (16)	0.25, (8)	0.063, (8)	0.5, (16)
**6h**	0.25, (2)	1, (4)	0.5, (4)	0.125, (4)	4, (2)
**6i**	0.25, (2)	1, (4)	0.5, (4)	0.25, (2)	2, (4)
**6j (TXA01182)**	0.063, (8)	0.5, (8)	0.25, (8)	0.125, (4)	2, (4)
**6l**	0.125, (4)	0.5, (8)	0.25, (8)	0.125, (4)	2, (4)
**-**	0.5	4	2	0.5	8

* Derived from K767, # clinical isolate.

Table S2. Ciprofloxacin and tigecycline potentiation in the presence of TXA01182 on resistant clinical isolates.

***P. aeruginosa* Strain**	**Ciprofloxacin MIC (µg/mL), (fold difference)**		**Tigecycline MIC (µg/mL), (fold difference)**		**Resistance Mechanisms**
	**No EPI**	**+ TXA01182 (6.25 µg/mL)**	**No EPI**	**+ TXA01182 (6.25 µg/mL)**	
AR-0229	32	8, (4)	128	8, (16)	*gyrA-T83I, nalC-G71E, mexR-V126Q*
AR-0232	4	1, (4)	16	8, (2)	*gyrA-T83I, nalC-G71E, mexR-V126Q*
AR-0234	4	1, (4)	16	8, (2)	*gyrA-T83I, nalC-G71E, mexR-V126Q*
AR-0239	32	16, (2)	16	8, (2)	*gyrA-T83I, nalC-G71E, mexR-V126Q*
AR-0244	64	16, (4)	64	8, (8)	*gyrA-T133H, nalC-G71E, mexR-V126Q*
AR-0246	32	16, (2)	64	16, (4)	*gyrA-T83I ,nalC-G71E, mexR-V126Q*
AR-0249	32	8, (4)	64	4, (16)	*gyrA-T83I, nalC-G71E*
AR-0264	32	8, (4)	16	2, (8)	*gyrA-D87Y, nalC-G71E*

Table S3. Frequency of resistance to Cefepime alone and in combination with TXA01182.

**Strain**	**Cefepime alone (4 µg/mL)**	**Cefepime (4 µg/mL) + TXA01182 (12.5 µg/mL)**
P. aeruginosa ATCC 27853	2.05×10^{-6}	$<1.04 \times 10^{-7}$
